# The Role of the *HOXA* Gene Family in Acute Myeloid Leukemia

**DOI:** 10.3390/genes10080621

**Published:** 2019-08-16

**Authors:** Si-Liang Chen, Zhe-Yuan Qin, Fang Hu, Yun Wang, Yu-Jun Dai, Yang Liang

**Affiliations:** 1 Department of Hematologic Oncology, Sun Yat-Sen University Cancer Center, Guangzhou 510060, China; 2 State Key Laboratory of Oncology in South China, Guangzhou 510060, China; 3 Collaborative Innovation Center for Cancer Medicine, Guangzhou 510060, China

**Keywords:** *HOXA* gene family, acute myeloid leukemia, transcription factor, prognostic value, molecular functions, bioinformatics integration analysis

## Abstract

The *HOXA* gene family is associated with various cancer types. However, the role of *HOXA* genes in acute myeloid leukemia (AML) have not been comprehensively studied. We compared the transcriptional expression, survival data, and network analysis of *HOXA*-associated signaling pathways in patients with AML using the ONCOMINE, GEPIA, LinkedOmics, cBioPortal, and Metascape databases. We observed that *HOXA2-10* mRNA expression levels were significantly upregulated in AML and that high *HOXA1-10* expression was associated with poor AML patient prognosis. The *HOXA* genes were altered in ~18% of the AML samples, either in terms of amplification, deep deletion, or elevated mRNA expression. The following pathways were modulated by *HOXA* gene upregulation: GO:0048706: embryonic skeletal system development; R-HSA-5617472: activation of *HOX* genes in anterior hindbrain development during early embryogenesis; GO:0060216: definitive hemopoiesis; hsa05202: transcriptional mis-regulation in cancer; and GO:0045638: negative regulation of myeloid cell differentiation, and they were significantly regulated due to alterations affecting the *HOXA* genes. This study identified *HOXA3-10* genes as potential AML therapeutic targets and prognostic markers.

## 1. Introduction

Acute myeloid leukemia (AML) is a heterogeneous hematological malignant cancer, characterized by the clonal expansion of myeloid blasts in the peripheral blood, bone marrow, and/or other tissues. It is the most prevalent acute leukemia amongst adults and the leading cause of leukemia-associated deaths in the United States each year [1]. An estimated 21,450 people will be diagnosed with AML in 2019, and 10,920 will die of this disease [2]. Reports show that the overall 5-year survival rate for AML patients has increased from 16% from 1991–1996 to 23% from 2003–2008 [3], followed by 28.4% from 2009–2015 [4]. In fact, about 45% of AML patients show a normal karyotype detected by conventional cytogenetics at diagnosis, whilst 97.3% display somatic mutations [5]. Therefore, there is an urgent need to identify reliable biomarkers, enabling early diagnosis and improved prognosis prediction of AML, in addition to developing novel targeted therapeutic strategies.

Mammals possess 39 *HOX* genes, which are arrayed in four linear clusters with 9 to 11 genes per cluster. Based on homologies, the genes are assigned to 13 paralogous groups. The *HOXA* gene family encodes proteins that contain the DNA-binding homeobox motif and controls the early patterns of embryo segmentation in addition to later developmental events [6]. *HOX* gene expression is typically inhibited in adults, although controlled reactivation is required for body repair and various homeostatic cellular processes [7,8], including hematopoiesis [9], wound healing [10], vascularization [10], as well as female reproductive tract development and fertility [11]. 

The expression of *HOX* genes is a common feature of AML, which is considered a disorder of the *HOX* pathway, leading to abnormal cell-renewal and leukemia development. In AML patients, the expression of *HOX* genes correlates most strongly to the translocation of the *MLL* gene [12]. *HOXA9*, in particular, has been shown to be a target of MLL fusion oncoproteins [13]. Several studies have shown that certain AML mutations are related to *HOX* gene expression and that high *HOXA* gene expression levels are usually accompanied by partial tandem *MLL* duplications [14]. Conversely, when *HOX* gene expression is low or absent, it is typically accompanied by *PML-RARA* and *RUNX1-RUNX1T1* gene fusions [15,16], as well as mutations of the *CEBPA* gene [17].

Therefore, the identification of *HOXA*-mediated oncogenes or tumor suppressors as potential mechanisms for predicting biomarkers may provide new therapeutic strategies for treating AML. The challenge lays in the fact that the differences in transcriptional levels, prognostic value, molecular functions, and biological processes for the majority of *HOXA* genes are yet to be elucidated in the context of AML disease. In this study, we expanded the knowledge on AML pathology by interrogating several universally-acknowledged databases to comprehensively analyze the relationship between *HOXA* subtypes and the pathogenesis and progression of AML.

## 2. Materials and Methods

### 2.1. Ethics Statement

This study was approved by the Academic Committee of the Sun Yat-Sen University Cancer Center, and it was carried out in accordance with the principles of the Helsinki Declaration. All the data analyzed within this study were retrieved from published literature, for which we confirm that written informed consent was obtained.

### 2.2. ONCOMINE Database Analysis

We analyzed *HOXA* gene transcription levels in different cancers using gene expression array datasets within ONCOMINE (https://www.oncomine.org), an online cancer microarray database [18]. *HOXA* mRNA expression levels in tumor specimens were compared with the levels in normal controls, and *P* values were obtained using the Students’ *t*-test. The cut-off *P* value and fold change were defined as 0.01 and 2, respectively. Genes co-expressed with *HOXA* genes were analyzed in the Cancer Genome Atlas (TCGA) for Leukemia, using the ONCOMINE database. Genes with a correlation value >0.4 were selected for further analysis.

### 2.3. The Gene Expression Profiling Interactive Analysis (GEPIA) Dataset

GEPIA [19] (http://gepia.cancer-pku.cn) is a newly developed interactive web server that enables users to perform expression analyses (such as survival and differential analyses) at the subtype level. GEPIA can be used to analyze gene and isoform expression by performing a comparison to TCGA and GTEx data. As such, we validated the differential expression of *HOXA* genes in AML and healthy donor samples.

### 2.4. The LinkedOmics Dataset

LinkedOmics [20] (http://www.linkedomics.orglogin.php) is a publicly-available portal comprising multiomics data from all 32 TCGA cancer types. It can be used to access, analyze, and compare multiomics data within and across tumor types. We performed a prognosis analysis for the *HOXA* gene family using the LinkedOmics AML databases.

### 2.5. TCGA Data and the cBioPortal

The cBioPortal [21] (http://www.cbioportal.org/) for cancer genomics is an open-access, open-source resource designed for the interactive exploration of multidimensional cancer genomics datasets. It supports and stores data relating to nonsynonymous mutations, DNA copy-number, mRNA and microRNA expression, protein-level and phosphoprotein level, DNA methylation, and de-identified clinical data. The cBioPortal online tool enabled us to calculate the frequency of gene alterations, mRNA expression z-scores (RNA Seq V2 RSEM), and *HOXA* family gene correlations.

### 2.6. Functional Enrichment and Bioinformatics Analysis

The Metascape [22] (http://metascape.org) online database integrates over forty bioinformatics knowledge bases enabling the extraction of abundant annotations, as well as the identification of enriched pathways and the construction of protein–protein interaction networks from lists of gene and protein identifiers. Using the Gene Ontology (GO) and the Kyoto Encyclopedia of Genes and Genomes (KEGG) tools within Metascape, we analyzed a genes list containing *HOXA* genes to identify the most frequently-altered linked genes.

## 3. Results

### 3.1. The Transcriptional Expression of HOXA Genes in Leukemia Patients

**Figure 1:**
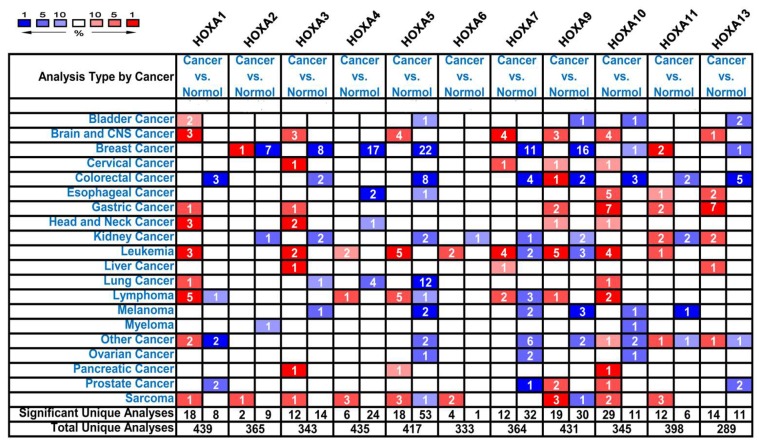
The transcription levels of *HOXA* genes in different types of cancers.

*HOXA* genes have been identified in the human genome. We used the ONCOMINE databases to compare the transcriptional expression of *HOXA* genes within tumorigenic and healthy control samples (Figure 1). ONCOMINE analysis revealed that the mRNA expression of *HOXA1*, *HOXA3*, *HOXA4*, *HOXA5*, *HOXA6*, *HOXA7*, *HOXA9*, and *HOXA10* was upregulated in patients with leukemia (Table 1). The analysis revealed that compared to healthy tissues, *HOXA1* mRNA was overexpressed 3.45-fold in AML [23] and 10.08-fold in acute adult T-cell leukemia/lymphoma (ATLL) [24]. In the Haferlach et al. dataset, *HOXA3* mRNA expression was upregulated in AML (fold change = 2.23) and in pro-B acute lymphoblastic leukemia (ALL) (fold change = 3.98), compared to healthy controls [25]. Moreover, Haferlach and colleagues showed that *HOXA4* was elevated in AML (fold change = 2.07) compared to the control samples [25]. Meanwhile, Andersson et al. reported that *HOXA4* was overexpressed in pro-B ALL (fold change = 8.08) [26]. In the Haferlach et al. dataset [25], the expression of the following *HOXA* genes was higher in the cancerous samples compared to the healthy controls: (i) *HOXA5* in AML (fold change = 2.52) and pro-B ALL (fold change = 4.36); (ii) *HOXA6* expression in pro-B ALL (fold change = 2.01); (iii) *HOXA7* expression in AML (fold change = 2.03); (iv) *HOXA9* expression in AML (fold change = 2.99) and pro-B ALL (fold change = 7.21); and (v) *HOXA10* expression in AML (fold change = 2.17) and pro-B ALL (fold change = 4.80). Stegmaier et al. reported the elevated expression of *HOXA5* (fold change = 6.58), *HOXA9* (fold change = 53.05), and *HOXA10* (fold change = 8.48) in AML [27]. In the Choi et al. dataset, *HOXA10* was upregulated in ATLL with a fold change of 3.95 [24]. According to the ONCOMINE analysis, no significant differences were observed in the mRNA expression levels of *HOXA2*, *HOXA11*, and *HOXA13* between leukemic and healthy control samples.

**Figure 2:**
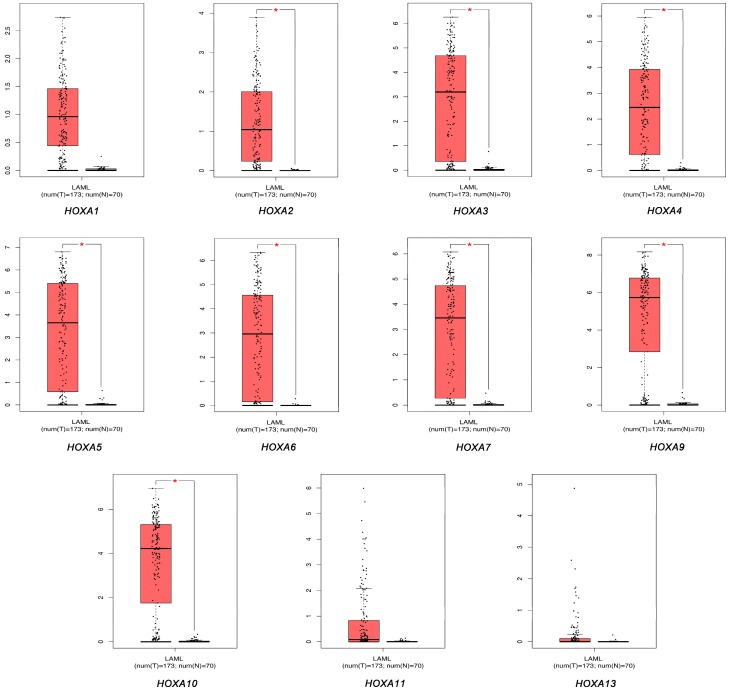
The expression of HOXA genes in acute myeloid leukemia (AML) and healthy control samples.

We used the GEPIA dataset to compare the mRNA expression levels of *HOXA* genes between leukemic and healthy control samples. The results indicated that the expression levels of *HOXA2*, *HOXA3*, *HOXA4*, *HOXA5*, *HOXA6*, *HOXA7*, *HOXA9*, and *HOXA10* were significantly increased in human AML patients compared to those in normal samples (*P* < 0.05), whilst the expression levels of *HOXA1*, *HOXA11*, and *HOXA13* were not significantly different (Figure 2).

**Figure 3:**
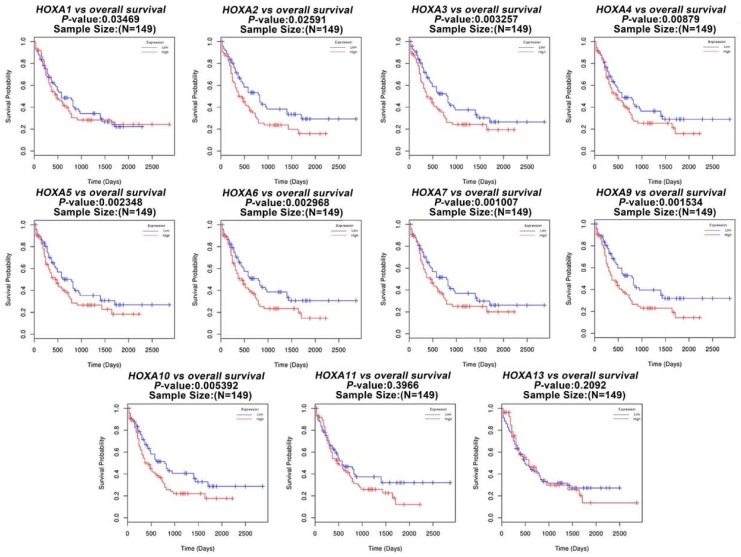
The prognostic value of the expression levels of *HOXA* genes in AML patients.

Next, we studied the role of *HOXA* genes in the survival of patients with AML. Using the LinkedOmics databases, we performed a prognosis analysis of *HOXA* genes in AML patients. The results revealed that the increased expression levels of *HOXA1*, *HOXA2*, *HOXA3*, *HOXA4*, *HOXA5*, *HOXA6*, *HOXA7*, *HOXA9*, and *HOXA10* were significantly correlated with poor overall survival (OS, *P* < 0.05) in all of the AML patients (Figure 3). Therefore, overexpression of *HOXA1-10* may represent a poor prognostic factor for AML.

### 3.2. Genetic Alteration and Correlations of HOXA Genes in AML

**Figure 4:**
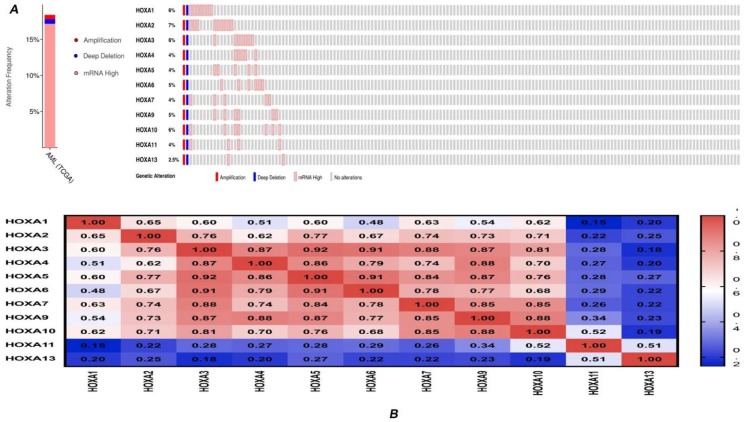
Genetic alteration and correlations of *HOXA* genes in AML. (**A**) Genetic alteration of *HOXA* genes in AML. (**B**) Genetic correlations of *HOXA* genes in AML.

Using the cBioPortal online tool and the ‘TCGA Provisional’ database for AML, we obtained information on genetic alterations in *HOXA* genes and identified any correlations between genes. *HOXA* genes were altered in 30/163 (18%) of AML patient samples (Figure 4A). The types of genetic alterations included amplification, deep deletion, and mRNA overexpression. In addition, cBioPortal was employed to analyze the expression of *HOXA* genes in AML (using mRNA sequencing [RNA-seq] version V2 RSEM), and we calculated the interactions between specific *HOXA* genes (including the Pearson’s correlation). The results revealed a significant positive correlation between any two *HOXA* family genes members with the exception of *HOXA1* and *HOXA11* (Pearson = 0.15, *P* = 0.0543) (Figure 4B).

### 3.3. Predicted Functions and Pathway Enrichment Analysis of HOXA Genes in AML Patients

**Figure 5:**
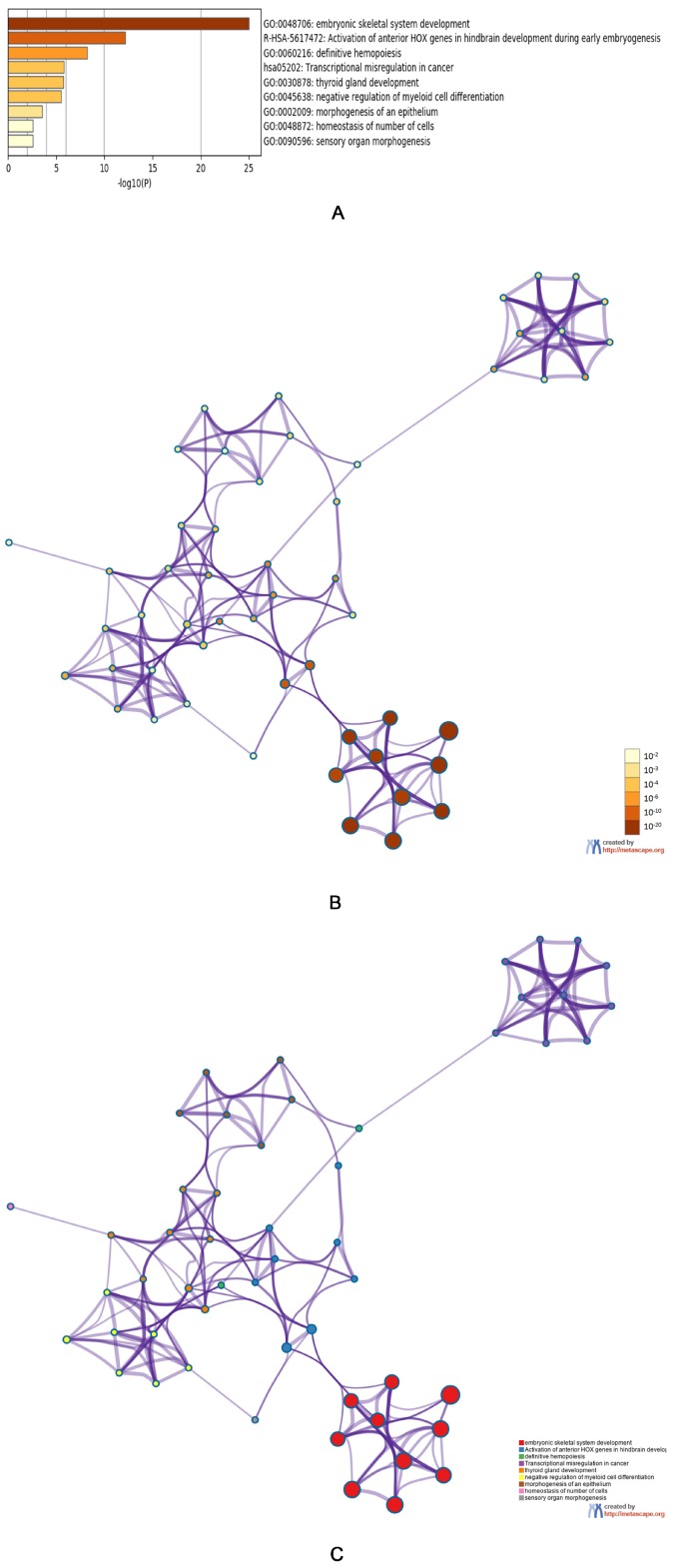
The functions of *HOXA* genes and genes significantly associated with *HOXA* genes alterations. (**A**) Heatmap of the Gene Ontology (GO) and Kyoto Encyclopedia of Genes and Genomes (KEGG) enriched terms colored by *P*-values. (**B**) Network of GO and KEGG enriched terms colored by *P*-value. (**C**) Network of GO and KEGG enriched terms colored by clusters.

Genes that were co-expressed in conjunction with *HOXA* genes were analyzed in the ‘TCGA Leukemia’ dataset using the ONCOMINE database. We found that the expression of *HOXA* genes was positively correlated with the upregulation of the following genes: *TSLP*, *HOXB2*, *HOXB3*, *HOXB4*, *LOC404266*, *HOXB6*, *NKX2-3*, *PBX3*, *C10orf140*, *LOC100271722*, *LOC100289444*, *MEIS1*, *CPNE8*, *LOC400931*, *SDSL*, *EMR1*, and *HOXB7*. We subsequently compiled a list of the expressed *HOXA* genes and the most frequently altered linked genes, prior to analyzing this gene list using the GO and KEGG tools in Metascape (Figure 5A–C). We found that the following processes were affected by *HOXA* gene alterations: GO:0048706: embryonic skeletal system development; R-HSA-5617472: activation of *HOX* genes in anterior hindbrain development during early embryogenesis; GO:0060216: definitive hemopoiesis, hsa05202: transcriptional misregulation in cancer; GO:0030878: thyroid gland development; GO:0045638: negative regulation of myeloid cell differentiation; GO:0002009: morphogenesis of epithelium; GO:0048872: homeostasis of several cell types; and GO:0090596: sensory organ morphogenesis.

## 4. Discussion

*HOX* genes belong to a family of transcription factor-encoding genes that share a DNA-binding homeobox domain [6]. In addition to their role in determining the characteristics of various body segments during embryogenesis, *HOX* genes also control the differentiation and regeneration of hematopoietic stem cells and precursor cells. The genes of the *HOXA* cluster, as well as the smaller *HOXB* family, are especially highly transcribed in hematopoietic precursor cells, and their expression gradually declines during maturation [28]. Dysregulation of members of the HOXA gene family has been reported in a variety of cancers, including glioblastoma [29], hepatocellular carcinoma [30], prostate cancer [31], gastric cancer [32], epithelial ovarian cancer [33], breast cancer [34], and leukemia [35,36,37,38]. Although the role of *HOXA* genes in the development of several cancers is well documented, a bioinformatics analysis of this gene family has not been performed in AML patient samples.

Previous research has shown that AML patients displaying favorable cytogenetic characteristics exhibit lower levels of *HOX* gene expression, whilst poor AML prognosis cases are associated with the upregulation of these genes [39]. *HOXA* family members were co-expressed with *HOXB3*, *HOXB6*, *MEIS1*, and *PBX3* in AML, and the expression levels of these genes were highly correlated [39]. The same results were observed in our study. Furthermore, we found that the expression of *HOXA3-10* was elevated in AML samples compared to healthy tissues, and a higher expression of *HOXA3-10* was significantly associated with poor OS in AML. The cBioPortal data indicated significant positive correlations between any two *HOXA* gene members, particularly between any two of the *HOXA3-10* genes. In addition, we found that *HOXA* gene expression was positively correlated with the upregulation of *HOXB2*, *HOXB3*, *HOXB4*, *HOXB6*, *NKX2-3*, *PBX3*, and *MEIS1*, amongst others. This result was consistent with the aforementioned work of Drabkin et al. [39].

It is worth noting that a previous study identified *HOXA4* as the only prognostic gene in AML, the upregulation of which in patients with normal karyotypes was actually associated with longer-term survival [40]. This finding is contrary to our results. However, on closer inspection of the work, we became aware that only 52 of the 126 AML patients (enrolled in the study from 1985 to 1997) were retained for the final analyses. Moreover, the study in question did not consider *FLT3* and *MLL* duplications, stating that the impact of *HOXA4* on survival was independent of the white blood cell count and the presence of the *FLT3*-internal tandem duplication (ITD). In addition to the small sample size, the work of Grubach et al. failed to consider the prognostic stratification of AML due to the limited information available at the time of the study [40]. Moreover, AML treatment regimens have undergone considerable change since the publication of the study in question. In light of this, we adhere to the view that the role of *HOXA4* in the survival of AML patients remains unclear and we await the emergence of further large-scale sample studies involving the *HOXA* gene family.

Numerous studies describe the functional role of the *HOXA* gene in the pathogenesis of AML. For instance, it has been shown that the *KDM3B* gene exhibits potential tumor-suppressive activity in AML and it transcriptionally regulates *HOXA1* via retinoic acid response elements [41]. A study by Li and colleagues showed that *HOXA5* knockout significantly inhibits the proliferation of AML cells, whilst shRNA down-regulation of *HOXA5* may induce apoptosis and overcome drug resistance in leukemia cells [42]. In addition, the upregulation of *HOXA5* through H3K79 methylation may represent a potential mechanism for leukemia transformation via the *CALM-AF10* fusion gene [43]. 

*HOXA7* and *HOXA9* appear to be required for efficient in vitro myeloid immortalization via the replacement of leukemia-associated fusion proteins with MLL fusion proteins. The involvement of *HOXA7* and *HOXA9* in MLL-mediated transformation suggests that the MLL oncoprotein acts as an upstream constitutive activator that promotes myeloid transformation through a *HOX*-dependent mechanism [44]. 

Amongst the *HOXA* genes, *HOXA9* is the most studied in AML [35,45,46,47]. Using the RNA interference technique, *HOXA9* has been shown to inhibit *MLL* rearrangement in *MLL*-rearranged leukemias, suggesting that targeting *HOXA9* or its downstream signaling partners may present an effective therapeutic strategy [46]. Similar to *HOXA9*, *HOXA11* has been implicated in leukemia development, exemplified by a patient with juvenile myelomonocytic leukemia (JMML) carrying a *NUP98-HOXA11* fusion gene [48]. In addition, a novel *NUP98* partner gene for *HOXA13*, with an expression pattern similar to *HOXA9* in leukemic cell lines, implies that the NUP98-HOXA13 fusion protein, like the NUP98-HOXA9 fusion protein, may play a role in leukemia development via the same mechanism [49]. 

At present, the functional roles of *HOXA* genes in AML merit further elucidation.

A large body of evidence has shown that AML, like normal hematopoiesis, is initiated and maintained by a subpopulation of leukemic stem cells (LSCs) [50]. Studies have shown that *NPM-1*, *ASXL1*, and *KAT6A* are the most important upstream regulators of LSCs epigenetic marker genes, which mainly regulate *HOXA* genes, including *HOXA5*, *HOXA6*, *HOXA7*, *HOXA9*, and *HOXA10* [51]. All three of these upstream regulators have been found to be mutated in AML, and may be driving *HOXA* gene expressions [52]. The work of Jung et al. has implied that the overexpression of *HOXA* genes may be mediated by DNA hypomethylation, a core mechanism for LSC activity [51]. Another study has demonstrated that *NPM1c* facilitates *HOX/MEIS1* expression and that *HOX* genes support the leukemic state in *NPM1*-mutant AML [53]. 

In this study, we employed GO and KEGG analysis tools to determine how the expression of *HOXA* genes and the most frequently-altered linked genes were associated with AML initiation and prognosis. We concluded that a few pathways deserved closer attention in the future, including: GO:0048706: embryonic skeletal system development; R-HSA-5617472: activation of *HOX* genes in anterior hindbrain development during early embryogenesis; GO:0060216: definitive hemopoiesis; hsa05202: transcriptional misregulation in cancer; and GO:0045638: negative regulation of myeloid cell differentiation. The last pathway has attracted our attention.

*Bcor*, a repressor of *HoxA* genes in myeloid cells, plays an indispensable role in hematopoiesis by inhibiting myeloid cell proliferation and differentiation [54]. Up-regulation of the *HOXA* gene in *Bcor* deletion may at least partially result in prolonged clonal growth of *Bcor* mutant hematopoietic stem/progenitor cells in replantation experiments [54]. 

In clinical practice, *Venetoclax*, a *Bcl-2* protein inhibitor of apoptosis, has been approved by the FDA for first-line use in AML, in combination with azacytidine, decitabine, or low-dose cytarabine. Studies have shown that the significant overexpression of specific *HOXA* gene transcripts was detected in samples with high *Bcl-2* inhibitor sensitivity [55]. *Bcl-2* inhibition has been shown to be effective in inducing the apoptosis of progenitor cells in patients with high-risk myelodysplastic syndrome and secondary AML [56]. Therefore, the mechanism through which *Bcl*-2 inhibitors modulate *HOXA* gene expression deserves further investigation.

## 5. Conclusions

Our results collectively suggest that the increased expression of *HOXA3-10* genes may be associated with the occurrence of AML, and that the increased expression of *HOXA3-10* may serve as a molecular marker to identify AML patients with an adverse prognosis. Thus, *HOXA3-10* may represent a prognostic marker and potential therapeutic target to improve the diagnosis and treatment of AML.

## Figures and Tables

**Table 1:** The significant changes in *HOXA* expression in transcription level between different types of leukemia and normal samples (ONCOMINE Database).

Gene	Types of leukemia vs. Normal Samples	Fold Change	*P* Value	*t*-Test	Reference
**HOXA1**	Acute Myeloid Leukemia vs. Normal	3.451	1.95E-07	9.046	Valk [23]
	Acute Adult T-Cell Leukemia/Lymphoma vs. Normal	10.084	3.46E-05	7.407	Choi [24]
**HOXA2**	NA	NA	NA	NA	NA
**HOXA3**	Acute Myeloid Leukemia vs. Normal	2.230	2.48E-68	19.863	Haferlach [25]
	Pro-B Acute Lymphoblastic Leukemia vs. Normal	3.975	8.57E-19	11.883	Haferlach [25]
**HOXA4**	Acute Myeloid Leukemia vs. Normal	8.083	4.21E-06	6.951	Andersson [26]
	Pro-B Acute Lymphoblastic Leukemia vs. Normal	2.065	8.86E-16	10.006	Haferlach [25]
**HOXA5**	Acute Myeloid Leukemia vs. Normal	6.584	7.75E-05	5.292	Stegmaier [27]
	Acute Myeloid Leukemia vs. Normal	2.522	2.78E-38	14.237	Haferlach [25]
	Pro-B Acute Lymphoblastic Leukemia vs. Normal	4.359	1.35E-15	9.826	Haferlach [25]
**HOXA6**	Pro-B Acute Lymphoblastic Leukemia vs. Normal	2.012	2.30E-14	9.174	Haferlach [25]
**HOXA7**	Acute Myeloid Leukemia vs. Normal	2.027	1.40E-67	19.782	Haferlach [25]
**HOXA9**	Acute Myeloid Leukemia vs. Normal	53.046	3.68E-07	9.445	Stegmaier [27]
	Acute Myeloid Leukemia vs. Normal	2.483	2.88E-33	13.493	Haferlach [25]
	Acute Myeloid Leukemia vs. Normal	2.986	9.55E-58	17.841	Haferlach [25]
	Pro-B Acute Lymphoblastic Leukemia vs. Normal	7.209	1.32E-20	12.798	Haferlach [25]
**HOXA10**	Acute Myeloid Leukemia vs. Normal	8.483	8.82E-06	7.123	Stegmaier [27]
	Acute Adult T-Cell Leukemia/Lymphoma vs. Normal	3.946	4.70E-05	4.888	Choi [24]
	Acute Myeloid Leukemia vs. Normal	2.174	1.30E-40	14.641	Haferlach [25]
	Pro-B Acute Lymphoblastic Leukemia vs. Normal	4.802	7.72E-19	11.645	Haferlach [25]
**HOXA11**	NA	NA	NA	NA	NA
**HOXA13**	NA	NA	NA	NA	NA

Abbreviations: NA, not available.
